# Establishment of a Novel Fluorescence-Based Method to Evaluate Chaperone-Mediated Autophagy in a Single Neuron

**DOI:** 10.1371/journal.pone.0031232

**Published:** 2012-02-07

**Authors:** Takahiro Seki, Ken-ich Yoshino, Shigeru Tanaka, Eisuke Dohi, Tomoya Onji, Kazuhiro Yamamoto, Izumi Hide, Henry L. Paulson, Naoaki Saito, Norio Sakai

**Affiliations:** 1 Department of Molecular and Pharmacological Neuroscience, Graduate School of Biomedical Sciences, Hiroshima University, Hiroshima, Japan; 2 Department of Neurology, University of Michigan, Ann Arbor, Michigan, United States of America; 3 Laboratory of Molecular Pharmacology, Biosignal Research Center, Kobe University, Kobe, Japan; University of Pittsburgh, United States of America

## Abstract

**Background:**

Chaperone-mediated autophagy (CMA) is a selective autophagy-lysosome protein degradation pathway. The role of CMA in normal neuronal functions and in neural disease pathogenesis remains unclear, in part because there is no available method to monitor CMA activity at the single-cell level.

**Methodology/Principal Findings:**

We sought to establish a single-cell monitoring method by visualizing translocation of CMA substrates from the cytosol to lysosomes using the HaloTag (HT) system. GAPDH, a CMA substrate, was fused to HT (GAPDH-HT); this protein accumulated in the lysosomes of HeLa cells and cultured cerebellar Purkinje cells (PCs) after labeling with fluorescent dye-conjugated HT ligand. Lysosomal accumulation was enhanced by treatments that activate CMA and prevented by siRNA-mediated knockdown of LAMP2A, a lysosomal receptor for CMA, and by treatments that inactivate CMA. These results suggest that lysosomal accumulation of GAPDH-HT reflects CMA activity. Using this method, we revealed that mutant γPKC, which causes spinocerebellar ataxia type 14, decreased CMA activity in cultured PCs.

**Conclusion/Significance:**

In the present study, we established a novel fluorescent-based method to evaluate CMA activity in a single neuron. This novel method should be useful and valuable for evaluating the role of CMA in various neuronal functions and neural disease pathogenesis.

## Introduction

In neurons, removal of misfolded proteins by protein degradation systems is important for various neuronal functions and survival. There are two major protein degradation systems: the ubiquitin-proteasome system (UPS) and the autophagy-lysosome system, which primarily degrade short-lived and long-lived proteins, respectively. The autophagy-lysosome system consists of three pathways: macroautophagy, microautophagy and chaperone-mediated autophagy (CMA). In neurodegenerative diseases, age-related decline in intracellular protein degradation is considered to cause accumulation and aggregation of misfolded protein in neurons, leading to neurodegeneration [1], [2]. This accumulation triggers additional impairment of protein degradation systems, resulting in further accumulation of misfolded proteins and additional neurodegeneration [3]. Accumulating evidence has revealed that UPS and macroautophagy are important for the clearance of misfolded proteins in neurons and are related to the pathogenesis of some neurodegenerative diseases [4], [5]. CMA is involved in the degradation of approximately 30% of cytosolic proteins and oxidized proteins [6], [7], suggesting that CMA contributes to protein quality control in neurons. Although there are several reports concerning CMA in Parkinson's disease [8], [9], [10], [11] and Alzheimer's disease [12], [13], the role of CMA in various neuronal functions and in other neurodegenerative diseases remains unknown. In neurons, CMA has been less studied than UPS and macroautophagy in part because there is neither an available protein marker to monitor CMA activity nor a method to evaluate CMA activity at the single-cell level. To overcome this problem, we sought to establish a novel fluorescence-based method to monitor CMA activity at the single cell level and to evaluate CMA activity in primary cultured neurons.

Spinocerebellar ataxia type 14 (SCA14) is an autosomal dominant neurodegenerative disorder that is clinically characterized by symptoms of cerebellar dysfunction and is caused by missense or deletion mutations in the *PRKCG* gene encoding protein kinase Cγ (γPKC) [14], [15], [16]. We previously demonstrated that mutant versions of γPKC form aggregates in cultured cells [17] and mouse primary cultured Purkinje cells (PCs) [18], leading to impairment of UPS and apoptotic cell death [19], as in other neurodegenerative diseases. We have also shown that mutant γPKC induces improper development of PC dendrites in an aggregate-independent manner [18]. Furthermore, mutant γPKC has aberrant kinase activities, higher basal activity in the cytosol and lower activity at the plasma membrane when activated [20], [21]. These findings suggest that mechanisms other than aggregation could participate in the neurodegeneration of cerebellar Purkinje cells in SCA14. To clarify the aggregate-independent mechanism, we used our newly established method for monitoring CMA to investigate whether mutant γPKC affects CMA activity in primary cultured PCs.

## Results

### Single-cell monitoring of CMA activity by visualizing lysosomal accumulation of a CMA substrate using the HaloTag system

**Figure 1:**
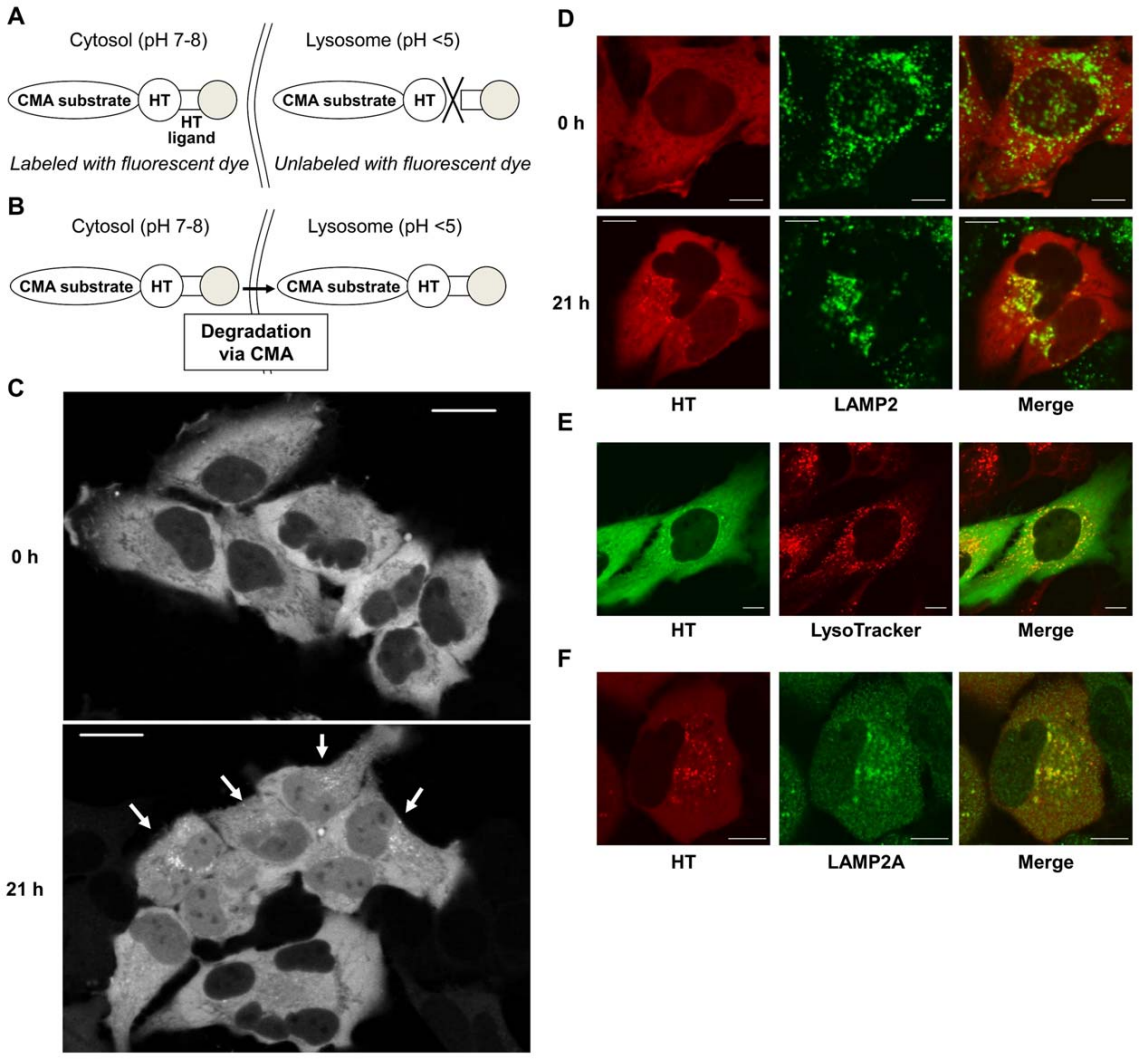
Visualization of CMA substrate translocation from cytosol to lysosomes using the HaloTag (HT) system. (*A*) Schematic illustration of CMA substrate-HT labeling with the HT ligand fused to fluorescent dye (gray circle). CMA substrate-HT in cytosol can be labeled with fluorescent dye since the HT reaction occurs at neutral pH. In contrast, CMA substrate-HT in lysosomes cannot be labeled since the HT reaction does not occur at acidic pH. (*B*) Schematic illustration of CMA substrate-HT translocation from cytosol to lysosome. Because the fluorescent dyes used in this study (TMR and OG) are not quenched at acidic pH, labeled CMA substrates can be visualized after translocation to lysosomes. (*C*) Representative fluorescent images of TMR-labeled GAPDH-HT immediately after (0 h, upper) and 21 h after labeling (21 h, lower) with TMR-HT ligand in HeLa cells. Arrows on the lower image indicate the cells with obvious GAPDH-HT dots. Bar = 20 µm. (*D*) Representative GAPDH-HT fluorescence (left), LAMP2 immunostaining (center) and merged (right) images of HeLa cells immediately after (0 h, upper panels) and 21 h after labeling (21 h, lower panels) with TMR-HT ligand. Bar = 5 µm. (*E*) Representative GAPDH-HT fluorescence (left), LysoTracker red fluorescence (center) and merged (right) images of HeLa cells 21 h after labeling with OG-HT ligand. Bar = 5 µm. (*F*) Representative GAPDH-HT fluorescence (left), LAMP2A immunostaining (center) and merged (right) images of HeLa cells 21 h after labeling with TMR-HT ligand. Bar = 5 µm.

In the process of CMA, cytosolic substrate proteins for CMA are recognized by heat shock cognate protein 70 (Hsc70), a molecular chaperone. The substrates are then transferred to the lysosome, where they are translocated through lysosome-associated membrane protein type 2A (LAMP2A) and subsequently degraded by lysosomal proteases [6]. Translocation of CMA substrates from the cytosol to lysosomes is a crucial step in CMA and thus indicates CMA activity. To monitor CMA activity at the single-cell level, we visualized the translocation of a CMA substrate from the cytosol to lysosomes using the HaloTag (HT) system [22]. HT-fused proteins produced in cells can be labeled by brief extracellular application of a fluorescently labeled HT ligand. When HT-fused proteins are exposed to an HT ligand, the HT covalently binds the HT ligand at neutral pH (cytosol and nucleus) but does not bind in the acidic lysosome, suggesting that only extralysosomal proteins are labeled by a fluorescent HT ligand (Fig. 1A). However, when HT-fused proteins are fluorescently labeled in the cytosol and translocated to lysosomes after further cultivation, the fluorescent labels (tetramethylrhodamine (TMR) and Oregon green (OG)) continue to fluoresce (Fig. 1B). Using this property, we sought to monitor the translocation of GAPDH, a well-known CMA substrate [6], from the cytosol to lysosomes.

GAPDH fused to HT (GAPDH-HT) was expressed in HeLa cells using adenoviral vectors (Fig. S1), and cells were incubated 10 min with a TMR-labeled HT ligand (TMR-HT ligand). Immediately after labeling, TMR-labeled GAPDH-HT was uniformly distributed in the cytoplasm, but 21 h of additional incubation led to dot-like cytoplasmic accumulations of GAPDH-HT in several cells (Fig. 1C). Immediately after labeling, GAPDH-HT did not colocalize with immunostained LAMP2, a lysosomal marker protein (Fig. 1D, upper), suggesting that only extralysosomal GAPDH-HT was labeled with the TMR-HT ligand. In contrast, the dot-like accumulations of GAPDH-HT after further incubation strongly colocalized with LAMP2 (Fig. 1D, lower). Furthermore, these dot-like accumulations of GAPDH-HT, labeled with OG-HT ligand, strongly colocalized with LysoTracker-red, a fluorescent lysosomal marker (Fig. 1E). These results indicate that translocation of GAPDH-HT to lysosomes can be visualized using the HT system.

**Figure 2:**
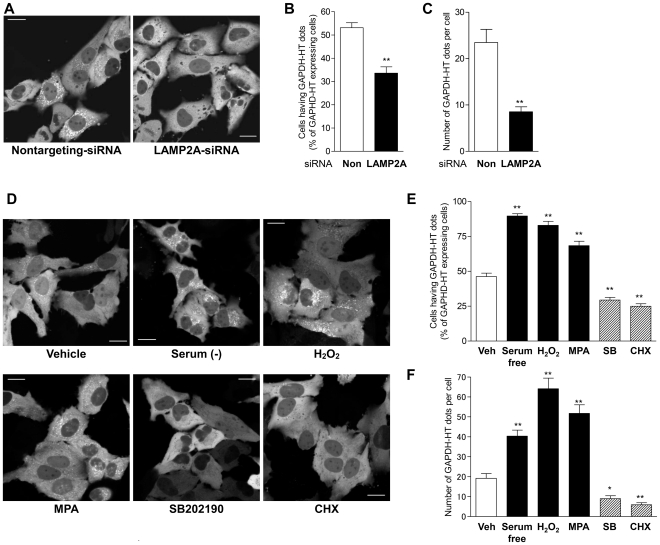
Lysosomal translocation of GAPDH-HT reflects CMA activity in HeLa cells. (*A*) Representative fluorescent images of GAPDH-HT 21 h after labeling with TMR-HT ligand in HeLa cells transfected with nontargeting-siRNA (left) or LAMP2A-siRNA (right). Bar = 20 µm. (*B,C*) Quantitative analyses of GAPDH-HT lysosomal translocation in cells transfected with nontargeting (Non)- and LAMP2A-siRNA. (*B*) Cells having more than 5 dots of GAPDH-HT were classified as GAPDH-HT dot-positive cells. We counted the number of GAPDH-HT dot-positive cells in 50–70 GAPDH-HT-expressing cells. (*C*) We assessed the number of GAPDH-HT dots per cell. The percentage of GAPDH-HT dot-positive cells and the number of GADPH-HT dots per cell were significantly decreased by siRNA-mediated LAMP2A-knockdown. ** p<0.001 vs cells treated with nontargeting-siRNA (unpaired *t*-test, n = 16 in *B*, n = 57 for nontargeting-siRNA and n = 74 for LAMP2A-siRNA in *C*). (*D*) Representative fluorescent images of GAPDH-HT taken 21 h after labeling with the TMR-HT ligand in HeLa cells treated with vehicle (0.1% DMSO, 0.1% methanol, left upper), serum free medium (0.1% DMSO, 0.1% methanol, center upper), H_2_O_2_ (100 µM, right upper), mycophenolic acid (MPA; 10 µM, left lower), SB202190 (20 µM, left center) or cycloheximide (CHX; 20 µg/ml, right lower). Bar = 20 µm. (*E,F*) Quantitative analyses of GAPDH-HT lysosomal translocation in cells treated with CMA activators or inhibitors the percentages of GAPDH-HT dot-positive cells (*E*) and the number of GAPDH-HT dots per cell (*F*). Percentages of GAPDH-HT dot-positive cells and the numbers of GAPDH-HT dots per cell were significantly increased by CMA activators (serum free medium, H_2_O_2_ and MPA), while they were significantly decreased by CMA inhibitors (SB202190 and CHX). * p<0.01, ** p<0.001 vs cells treated with vehicle (unpaired *t*-test, n = 12 for cells treated with vehicle, n = 8 for cells treated with CMA activators and inhibitors in *E*, n = 96 for vehicle, n = 61 for serum free, n = 45 for H_2_O_2_, n = 58 for MPA, n = 52 for SM202190 and n = 41 for CHX in *F*).

Next, we examined whether this translocation is mediated by CMA. Mammalian LAMP2, which is localized in the lysosomal membrane, consists of three splicing isoforms (LAMP2A, B, C) that are alternatively spliced in their carboxyl termini [23]. Among the isoforms, only LAMP2A is involved in CMA as a lysosomal receptor for CMA substrates [24]. Most dots of GAPDH-HT were colocalized with LAMP2A-specific antibody staining (Fig. 1F), indicating that GAPDH-HT accumulates in lysosomes containing LAMP2A. Next, we examined whether lysosomal translocation of GAPDH-HT would occur in HeLa cells with siRNA-mediated knockdown of LAMP2A. We designed a LAMP2A-siRNA that targets an RNA sequence corresponding to the carboxyl terminus of LAMP2A, which is unique to LAMP2A and absent in other LAMP2 isoforms. Transfection with LAMP2A-siRNA nearly abolished the expression of LAMP2A while only partially decreasing the amount of total LAMP2 (Fig. S2A,B), suggesting that LAMP2A-siRNA specifically targets and decreases LAMP2A among the three LAMP2 isoforms. Compared to nontargeting control siRNA, LAMP2A-siRNA significantly inhibited the dot-like accumulation of GAPDH-HT in transfected cells (Fig. 2A–C). Furthermore, CMA is reported to be activated by various stimuli, including long-term serum deprivation [25], oxidative stress [7] and mycophenolic acid (MPA), which decrease intracellular GTP levels [26]. Therefore, we monitored the translocation of GAPDH-HT in the presence or absence of these treatments for 21 h following labeling with the TMR-HT ligand. The proportion of cells showing dot-like accumulations of GAPDH-HT was significantly increased by serum deprivation, 100 µM H_2_O_2_ and 10 µM MPA (Fig. 2D,E). In addition, the number of dots per cell was also increased by these treatments (Fig. 2F). In contrast, CMA is inhibited by the inhibitors of p38 mitogen activated protein kinase (MAPK) and by cycloheximide [27]. Indeed, lysosomal translocation of GAPDH-HT was significantly inhibited by 20 µM SB202190, a p38 MAPK inhibitor and 20 µg/ml cycloheximide (Fig. 2D–F). These results indicate that CMA activity can be assessed by monitoring lysosomal translocation of GAPDH-HT, representing a novel method to evaluate CMA in individual cells.

Since macroautophagy is considered a nonselective protein degradation pathway for cytosolic proteins, it is possible that lysosomal accumulation of GAPDH-HT is mainly mediated by macroautophagy, not by CMA. To exclude this possibility, we examined lysosomal translocation of GAPDH-HT in cells treated with siRNA against Atg5, which is essential for macroautophagy. We confirmed that Atg5-siRNA decreased the amount of Atg5-Atg12 complex and macroautophagy activity (Fig. S2). We also examined the effects of 3-methyladenine (3-MA), which suppresses macroautophagy by inhibiting type III phosphatidylinositol 3-kinase [28], on translocation of GAPDH-HT. Lysosomal translocation of GAPDH-HT was not affected by Atg5-siRNA or 10 mM 3-MA (Fig. S3A–D). Moreover, GAPDH-HT accumulated in LAMP2A-positive lysosomes in embryonic fibroblast (MEF) cells from Atg5-knockout (KO) mice (Fig. S3E). These findings suggest that the lysosomal accumulation of GAPDH-HT is not mediated by macroautophagy.

Macroautophagy is activated to compensate for CMA impairment by LAMP2A knockdown [29]. In the present study, LAMP2A-siRNA increased the dot-like immunostaining of LC3 and the amount of LC3-II, which is specifically localized to autophagosomes [30] (Fig. S2), confirming that LAMP2A-siRNA activated macroautophagy in HeLa cells. Interestingly, LAMP2A-siRNA increased the abundance of the Atg5-Atg12 complex detected with the anti-Atg5 antibody (Fig. S2), suggesting that CMA affects macroautophagy activity by regulating Atg5-Atg12 complex levels. If GAPDH-HT accumulated in lysosomes by macroautophagy, this accumulation would be stimulated by LAMP2A-siRNA. However, accumulation was inhibited by LAMP2A-siRNA. Moreover, dots of GAPDH-HT rarely colocalized with dot-like immunostaining of LC3 even in the presence of CMA activators, while they strongly colocalized with or were surrounded by LAMP2A immunoreactivity (Fig. S4). Collectively, these results indicate that the lysosomal translocation of GAPDH-HT is mainly mediated by CMA and not by macroautophagy. We therefore consider the observation of GAPDH-HT lysosomal translocation a novel and valid method to evaluate CMA activity at a single cell level.

### Single-cell monitoring of CMA activity in primary cultured cerebellar Purkinje cells

**Figure 3:**
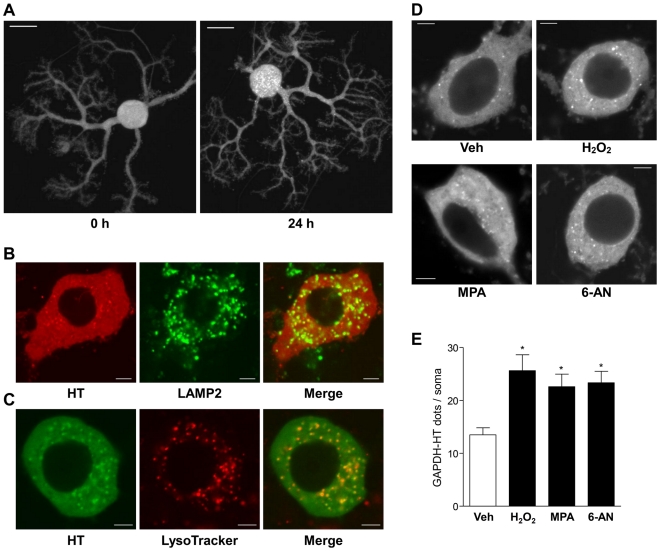
Lysosomal translocation of GAPDH-HT in primary cultured PCs. (*A*) Representative fluorescent images of TMR-labeled GAPDH-HT immediately after (0 h, left) and 24 h after labeling (24 h, right) with TMR-HT ligand in primary cultured PCs. Images were projected from a Z-stack of images obtained by confocal laser microscopy. Bar = 20 µm. (*B*) Representative GAPDH-HT fluorescence (left), LAMP2 immunostaining (center) and merged (right) images of PC somata 21 h after labeling with TMR-HT ligand. Images were taken from the center of the Z-stack. Bar = 5 µm. (*C*) Representative GAPDH-HT fluorescence (left), LysoTracker red fluorescence (center) and merged (right) images of PC somata 21 h after labeling with TMR-OG ligand. Bar = 5 µm. (*D*) Representative fluorescent images of GAPDH-HT in PC somata treated with vehicle (0.1% DMSO, 0.1% methanol, left upper), H_2_O_2_ (100 µM, right upper), mycophenolic acid (MPA; 10 µM, left lower) and 6-aminonicotinamide (6-AN; 1 mM, right lower). Bar = 5 µm. (*E*) Quantitative analyses of lysosomal translocation of GAPDH-HT in PC somata treated with CMA activators. Dots of GAPDH-HT in each PC soma were counted in the center image from the Z-stack. Numbers of GAPDH-HT dots were significantly increased in the presence of CMA activators (H_2_O_2_, MPA and 6-AN). * p<0.01 vs PCs treated with vehicle (unpaired *t*-test, n = 60 for cells treated with vehicle, n = 30 for cells treated with CMA activators).

To investigate whether CMA activity could be assessed in primary cultured neurons by the lysosomal-translocation method, we expressed GAPDH-HT selectively in primary cultured cerebellar Purkinje cells using adenoviral vectors (Fig. S1) [18]. Similar to HeLa cells, TMR-labeled GAPDH-HT was uniformly distributed throughout PC somata and dendrites immediately after labeling with the TMR-HT ligand. In contrast, most PCs had many cytoplasmic dots of GAPDH-HT 24 h after labeling, with dots being especially abundant in PC somata (Fig. 3A). These dots also colocalized with LysoTracker and LAMP2 immunoreactivity (Fig. 3B,C). In contrast to the results in HeLa cells, cytoplasmic dots were observed in almost all PC somata. Therefore, we quantitatively assessed CMA activity by counting the number of dots per PC soma. The number of GAPDH-HT dots in PC somata was significantly increased by the CMA activating stimuli, 100 µM H_2_O_2_, 10 µM MPA and 1 mM 6-aminonicotinamide (6-AN), which is also reported to activate CMA [27] (Fig. 3D,E). Furthermore, we attempted to investigate whether siRNA-mediated knockdown of LAMP2A inhibits lysosomal translocation of GAPDH-HT in primary cultured neurons, as seen in HeLa cells. For this purpose, we used cortical neurons, instead of Purkinje cells, because it was difficult to knockdown LAMP2A in a Purkinje cell-specific manner. We confirmed that knockdown of LAMP2A significantly inhibited lysosomal translocation of GAPDH-HT in primary rat cortical neurons (Fig. S5). These results suggest that the lysosomal-translocation method can be used to evaluate CMA activity in primary cultured neurons.

### SCA14 mutant γPKC impairs CMA in HeLa cells and primary cultured PCs

**Figure 4:**
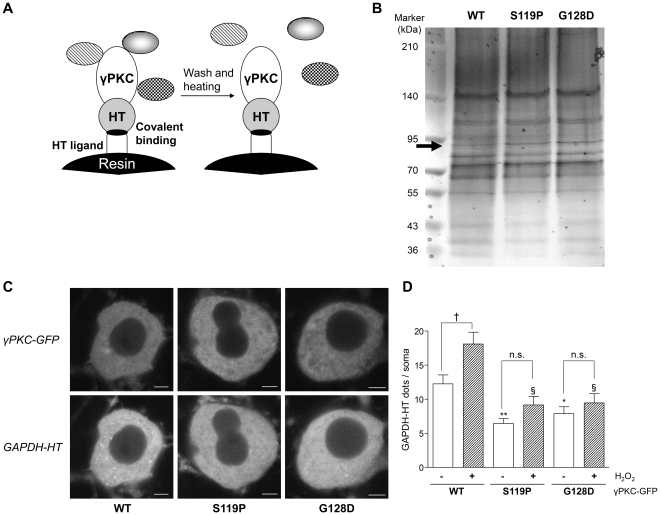
SCA14 mutant γPKC preferably binds with Hsc70 and suppresses CMA in primary cultured PCs. (*A*) Schematic illustration of pull-down assay using the HT system. Resin-conjugated HT ligands covalently bind to γPKC-HT and harvest γPKC-HT and γPKC-binding proteins from cell lysate. After washes and heat treatment, γPKC-binding proteins are released from the resin, but γPKC-HT itself is still resin-bound through the covalent linkage between HT and the HT ligand. This is a unique feature of the HT pull-down assay, in contrast to other pull-down assays using glutathione S-transferase or immunoprecipitation, and enables us to effectively analyze binding partners of the target protein. (*B*) Representative silver stained gel of pulled down proteins by the HT pull-down assay and subjected to SDS-PAGE. The HT pull-down assay was conducted using cell lysate from cerebellar primary cultures that expressed WT or mutant (S119P and G128D) γPKC-HT in PCs. The arrow indicates the protein band that was more strongly detected in pull-down samples from cell lysates expressing mutant γPKC-HT than in the WT. Mass spectrometric analysis revealed that this protein is Hsc70. Silver staining gel with pull-down, input and supernatant (Sup) samples is indicated in Figure S5A. (*C*) Representative fluorescent images of γPKC-GFP (upper panels) and GAPDH-HT (lower panels) in PC somata 21 h after labeling with TMR-HT ligand. PCs coexpressed GAPDH-HT and either WT (left), S119P (center) or G128D (right) mutant γPKC-GFP. We evaluated GAPDH-HT lysosomal translocation in PCs not displaying aggregation of mutant γPKC-GFP. Bar = 5 µm. (*D*) Quantitative analyses of GAPDH-HT lysosomal translocation in PC somata in the absence (open bars) or presence (hatched bars) of 100 µM H_2_O_2_. Mutant versions of γPKC-GFP (S119P and G128D) significantly decreased the number of GAPDH-HT dots (* p<0.05, ** p<0.001 vs PCs expressing WT γPKC-GFP without H_2_O_2_, unpaired *t*-test, n = 45, and § p<0.001 vs PCs expressing WT γPKC-GFP with H_2_O_2_, unpaired *t*-test, n = 45). While H_2_O_2_ significantly increased the number of GAPDH-HT dots in PCs expressing WT γPKC-GFP († p<0.01, unpaired *t*-test), it failed to increase the number of GAPDH-HT dots in PCs expressing mutant γPKC-GFP (n.s.: not significant (p>0.05), unpaired *t*-test).

Using an HT pull-down assay, we identified Hsc70 as a preferred binding protein for the SCA14 mutant γPKC in primary cultured PCs (Fig. 4A, B, Fig. S6A). This interaction was confirmed by HT pull-down assay in cultured cell line (Fig. S6B,C). Furthermore, 2-color fluorescence recovery after photobleaching (FRAP) and raster image correlation spectroscopy (RICS) analyses revealed that mutant γPKC-GFP interacted with TMR-labeled Hsc70-HT, thereby reducing the mobility of Hsc70-HT in living PCs (Table S1 and S2). As Hsc70 is involved in CMA [6], it is possible that mutant γPKC affects CMA activity in PCs. To explore this possibility, we evaluated lysosomal translocation of GAPDH-HT in PC somata coexpressing wild type (WT) or mutant γPKC-GFP without aggregation of the mutant protein. Whereas WT γPKC-GFP did not affect lysosomal translocation of GAPDH-HT compared with cells expressing GFP alone (14.4±1.6 dots/soma, n = 45), mutant γPKC-GFP significantly decreased the number of GAPDH-HT dots in PC somata (Fig. 4C, D). Similar results were obtained using HeLa cells coexpressing γPKC-GFP and GAPDH-HT (Fig. S7A,B). Furthermore, expression of mutant γPKC-GFP significantly increased the amount of another CMA substrate, myocyte enhancer factor 2D (MEF2D) (Fig. S7C,D) [10]. In addition, oxidative stress (100 µM H_2_O_2_) failed to increase the number of GAPDH-HT dots in PC somata expressing mutant γPKC-GFP but did significantly increase GAPDH-HT dots in PC somata expressing WT γPKC-GFP (Fig. 4D). These results suggest that mutant γPKC inhibits both basal and induced CMA activity in primary cultured PCs.

## Discussion

In contrast to macroautophagy, it remains unclear how CMA activity is regulated and how CMA relates to various physiological functions, even though several molecules involved in CMA have been discovered. While macroautophagy activity can be easily assessed by the amount and localization of LC3-II [30], [31], existing methods for evaluating CMA activity require special lysosome isolation techniques or radioisotopic analysis. In addition, these methods only reflect CMA activity for a large cell population [32], which has hampered a detailed understanding of CMA. In the present study, we succeeded in establishing a novel method to monitor CMA at a single-cell level. Our method enables simple and real-time observation of CMA activity in single cells and may represent a breakthrough for elucidating the regulation of CMA and the role of CMA in normal cellular functions and in disease pathogenesis.

We believe that GAPDH-HT accumulates in lysosome since dots of GAPDH-HT colocalize with LAMP2 immunoreactivity and LysoTracker red fluorescence (Fig. 1 D, E and Fig. 3 B, C). However, LAMP2A is frequently used as a marker of both late endosomes and lysosomes [33], and LysoTracker stains acidic organelles including late endosomes and lysosomes [34]. Therefore, we cannot exclude the possibility that GAPDH-HT accumulates in late endosomes as well as lysosomes. Recently, it has been demonstrated that late endosomes are involved in mammalian microautophagy, in which the delivery of cytosolic substrates to late endosomes is mediated by Hsc70, but not by LAMP2A [35]. Therefore, GAPDH-HT might partly accumulate in late endosomes via microautophagy, which is supported by the result that siRNA-mediated knockdown of LAMP2A did not completely inhibit the dot-like accumulation of GAPDH-HT (Fig. 2B,C). However, because this dot-like accumulation was highly sensitive to chemicals that affect CMA activity (Fig. 2D–F), CMA is likely more responsible for this accumulation than Hsc70-mediated microautophagy.

Reciprocal crosstalk has been reported between CMA and macroautophagy; when one is impaired, the other is activated to compensate [29], [36]. Similarly, in the present study siRNA-mediated LAMP2A knockdown activated macroautophagy (Fig. S2). However, it remains unclear how CMA blockage activates macroautophagy. Ubiquilin was identified as a candidate to regulate this crosstalk [37]; it is involved in the formation of autophagosomes in macroautophagy and is degraded both by macroautophagy and by CMA. Blockage of CMA would increase the amount of ubiquilin, leading to the activation of macroautophagy. In the present study, we showed that the amount of the Atg5-Atg12 complex, which is essential for autophagosome formation [38], was elevated by siRNA-mediated LAMP2A knockdown (Fig. S2), suggesting that this complex is involved in the crosstalk between macroautophagy and CMA. Unlike ubiquilin, Atg5 and Atg12 are not substrates for CMA since they do not have the KFERQ-like motif required for recognition by Hsc70 and degradation through CMA [6]. In this scenario, CMA would affect macroautophagy by regulating other proteins involved in Atg5 and Atg12 expression, degradation or complex formation.

In the present study, we evaluated CMA activity in HeLa cells and primary cultured PCs through dot-like accumulation of GAPDH-HT. Approximately 60% of HeLa cells expressed GAPDH-HT dots (Fig. 1C and 2), but almost all PCs had many dots in somata and dendrites (Fig. 3A). This difference suggests that CMA activity is higher and more important for removal of aberrant proteins in nonproliferative neurons than in proliferative cells. Although most dots were localized to PC somata, several dots were observed in PC dendrites (Fig. 3A). This suggests that CMA may participate in the development of dendritic structures or maintenance of dendritic functions. Further studies are necessary to elucidate the role of CMA in PC dendrites.

In the present study, we demonstrated that the SCA14 mutant γPKC preferentially interacts with Hsc70 and impairs CMA in primary cultured PCs. γPKC has three KFERQ-like motifs in its amino acid sequence (QLEIR at residues 162–166, LKLDN at 481–485 and NFDKF at 642–646). Therefore, it is possible that a strong interaction between mutant γPKC and Hsc70 hampers the binding of other CMA substrates to Hsc70 and thus inhibits their degradation. In the present study, 2-color FRAP analysis revealed that mutant γPKC-GFP increased the mobility of GAPDH-HT in living PCs (Table S1). This observation may be explained if Hsc70 were prevented from interacting with GAPDH, so that GAPDH would be released from a GAPDH-Hsc70 complex. Since CMA is involved in adaptation to stress conditions [7], [29], CMA impairment by mutant γPKC could exacerbate cellular vulnerability to various cellular stresses such as oxidative stress and contribute to neurodegeneration of PCs in SCA14.

We used GAPDH as a CMA substrate to monitor CMA activity, since it has been widely used for CMA studies [6], [7]. Although the lysosomal translocation of GAPDH-HT was significantly inhibited by mutant γPKC (Fig. 4 and Fig. S7A,B), it did not affect the total amount of GAPDH (Fig. S7C,D). Since GAPDH is a key enzyme in glycolysis, the amount of GAPDH might be strictly regulated by transcriptional level as well as degradation through CMA. Indeed, we found that total GAPDH amount was not affected by siRNA-mediated LAMP2A knockdown (Fig. S7E,F). Similar findings were also reported by Vogiatzi, et al [39]. Instead, we confirmed CMA inhibition by mutant γPKC through the significant increase of another CMA substrate, MEF2D (Fig. S7C,D), which is reported to be elevated by the knockdown of LAMP2A [10].

Several studies have demonstrated that CMA is involved in the pathogenesis of Parkinson's disease [8], [9], [10], [11] and Alzheimer's disease [12], [13]. Our novel CMA monitoring method can be available for further and precise elucidation of the involvement of CMA in neural function and in the pathogenesis of neurodegenerative diseases. Indeed, we succeeded to reveal the alteration of CMA activity by a mutant protein causing neurodegenerative disease in a single neuron level. Moreover, selective CMA degradation of expanded polyglutamine alleviates the pathology and phenotype in a mouse model of Huntington's disease [40]. Elucidation of CMA function would raise the possibility that CMA is a novel therapeutic target for neurodegenerative diseases. Our method is also available as a screening tool for chemicals that affect CMA activity, and thus could facilitate the discovery of novel CMA-targeted therapeutics for neurodegenerative diseases and help elucidate the role of CMA in normal and pathogenic processes.

## Materials and Methods

### Materials

Dulbecco's modified Eagle medium (DMEM), mycophenolic acid, SB202190 and MISSION siRNA universal negative control (nontargeting-siRNA) were obtained from Sigma-Aldrich. The SUMITOMO Nerve-Cell Culture System (Neuron culture medium and dissociation solutions) was from Sumitomo Bakelite (Tokyo, Japan). Penicillin/streptomycin solution, hydrogen peroxide and cycloheximide were from Nakalai Tesque (Kyoto, Japan). Tokyo Chemical Industry (Tokyo, Japan) supplied 6-aminonicotinamide. The HaloTag system (HaloTag vector and HaloTag ligand) was from Promega (Madison, WI). Fluorescein isothiocyanate (FITC)-conjugated donkey anti-rat IgG antibody was from Jackson ImmunoResearch Laboratories (West Grove, PA). Anti-human LAMP2 mouse monoclonal and anti-mouse LAMP2 rat monoclonal antibodies were from the developmental studies hybridoma bank (Iowa, IA). Anti-mouse LAMP2A rabbit polyclonal antibody and AlexaFluor 488 (Alexa488)-conjugated goat anti-mouse IgG and anti-rabbit IgG antibodies, normal goat serum (NGS), LysoTracker red, Lipofectamine RNAiMAX and Hank's balanced salt solution were from Invitrogen (Carlsbad, CA). Anti-human/mouse LAMP2A rabbit polyclonal antibody was from Abcam (Cambridge, UK). Glass-bottomed culture dishes (35-mm diameter) were from MatTek (Ashland, MA). Materials for supporting figures and tables were listed in Methods S1.

### Cell culture

HeLa cells were purchased from Riken Cell Bank (Tsukuba, Japan). HeLa cells were cultured in DMEM supplemented with 10% fetal bovine serum (FBS), 100 units/ml of penicillin and 100 µg/ml of streptomycin in a humidified atmosphere containing 5% CO_2_ at 37°C. Mouse cerebellar primary culture was prepared using SUMITOMO Nerve-Cell Culture System according to the manufacturer's protocol [18]. Briefly, cerebella of E14 embryos from pregnant ICR mice were dissociated using dissociation solutions and were cultured in the neuron culture medium for 21–30 days *in vitro* (DIV). This experiment was conducted according to the guideline of animal use and approved by the animal care and use committee of Hiroshima University (permit number: A08-68).

### Adenoviral vectors using a tetracycline (Tet)-regulated system

We constructed two types of adenoviral vectors to express the target proteins (GFP- and HT-fused proteins) under the regulation of tetracycline [41] (Fig. S1). The first vector expresses the tetracycline transactivator (tTA) under the control of the cytomegalovirus (CMV) promoter for HeLa cells (Ad-CMV-tTA) or the L7 promoter for primary cultured PCs (Ad-L7-tTA). The second vector expresses the target protein under the control of the TetOp minimal promoter, which is activated by tTA (Fig. S1). Use of the first and second vectors in combination enables cell type-specific expression of target proteins.

Ad-CMV-tTA, Ad-L7-tTA, Ad-TetOp-γPKC-GFP (wild type (WT), S119P and G128D mutant) and Ad-TetOp-GAPDH-HT were constructed using the AdEasy adenoviral vector system (Stratagene, La Jolla, CA) according to the manufacturer's protocol, as previously described [18].

### Observation of lysosomal translocation of GAPDH-HT proteins

HeLa cells (1×10^5^ cells/glass-bottomed culture dish) were infected with Ad-CMV-tTA (MOI of 10) and Ad-TetOp-GAPDH-HT (MOI of 10) 1 day after cell spread and cultured for an additional 2–3 days. Cells were incubated with culture medium containing 500 nM TMR-HT ligand for 10 min at 37°C, followed by 3 washes with culture medium. Cells were fixed with 4% paraformaldehyde in PBS immediately after labeling or after 21 h of cultivation. In some experiments, cells were treated with culture medium containing vehicle (0.1% DMSO and 0.1% methanol), CMA activator (serum deprivation, 100 µM H_2_O_2_ or 10 µM mycophenolic acid) or CMA inhibitor (20 µM SB202190 or 20 µg/ml cycloheximide). Lysosomal translocation of GAPDH-HT was assessed by its colocalization with LAMP2, LAMP2A or LysoTracker-red. LAMP2 and LAMP2A were immunostained with anti-human LAMP2 monoclonal antibody (diluted 1∶3000) and anti-LAMP2A rabbit polyclonal antibody (1∶200), respectively, followed by Alexa488-conjugated goat anti-mouse and anti-rabbit IgG antibodies (1∶500), respectively, as described previously [19]. To further confirm lysosomal localization of GAPDH-HT, it was labeled with OG, and cells were stained with 25 nM LysoTracker red, a lysosomal marker, for 30 min before fixation. Fluorescent images were obtained using a confocal microscope (LSM510META, Carl Zeiss, Oberkochen, Germany). Cells having more than 5 dots were classified as GAPDH-HT dot-positive cells. We quantitatively evaluate lysosomal accumulation of GAPDH-HT by the percentage of GAPDH-HT dot-positive cells in 50–70 GAPDH-HT expressing cells per culture. For further assessment of lysosomal accumulation, we counted the number of GAPDH-HT dots per 40–100 independent GAPDH-HT-expressing cells.

Cerebellar primary cultures (2×10^5^ cells/glass-bottomed culture dish) were infected with Ad-L7-tTA (MOI of 20) and Ad-TetOp-GAPDH-HT (MOI of 3) and/or Ad-TetOp-γPKC-GFP (MOI of 3) on DIV20–22 and cultured for 7 days. Living cells were incubated with 500 nM of TMR-HT ligand for 10 min at 37°C. After 4 washes with culture medium, cells were cultured for 21 h in the presence or absence of various compounds and fixed with 4% paraformaldehyde in PBS. LAMP2 was immunostained with anti-mouse LAMP2 rat monoclonal antibody (1∶100), followed by FITC-conjugated donkey anti-rat IgG (1∶400) antibody. Fluorescent images of whole PCs and PC somata were obtained as a Z-stack of images using a confocal microscope. We quantitatively evaluated lysosomal accumulation of GAPDH-HT by the number of cytoplasmic dots per PC soma. We counted the number in the center image of the Z-stack from somata of 30–60 independent PCs.

### Pull-down assay using the HaloTag system

A cerebellar primary culture (4×10^5^ cells/3.5 cm culture dish) was infected with Ad-L7-tTA at a multiplicity of infection (MOI) of 20 and Ad-TetOp-γPKC-HT (MOI of 3) on DIV14 and cultured for 14 days. Cells were harvested and lysed in lysis buffer (1% Triton X-100 in PBS containing 20 µg/ml of leupeptin, 1 mM phenylmethanesulfonyl fluoride (PMSF), 1 mM NaF, 100 nM Calyculin A). Cell lysates were cleared by centrifugation for 15 min at 15,000×g at 4°C and rotated with 30 µl of HaloLink resin (resin fused with HT ligand) for more than 8 h at 4°C. After 3 PBS washes of the resin, proteins bound to γPKC-HT were eluted in sample buffer (3% SDS, 2% 2-mercaptoethanol, 5% glycerol, 0.002% bromophenol blue, 92 mM Tris-HCl, pH 6.8) by heating (95°C for 3 min). Pulled down proteins, input (10%) and supernatant (10%) after pull-down were subjected to SDS-PAGE using gradient (5–20%) acrylamide gel, and the separated proteins were visualized by silver staining. Specific protein bands were excised from the gel and subjected to mass spectrometry analysis.

### siRNA

Nontargeting-siRNA was used as a negative control of siRNA transfection. siRNAs against human LAMP2A (sense: 5′-GGCAGGAGUACUUAUUCUAGU-3′, antisense: 5′-UAGAAUAAGUACUCCUGCCAA-3′) and human Atg5 (sense: 5′-CACUUUCAGAAGGUUAUGAGA-3′, antisense: 5′-UCAUAACCUUCUGAAAGUGCU-3′) were constructed by Hayashi-kasei (Osaka, Japan). siRNA (50 pmol) was transfected to HeLa cells 1 day after cell spread using Lipofectamine RNAiMAX. The culture medium was exchanged 4 h after transfection. To express GAPDH-HT in siRNA-transfected cells, adenoviral vectors were added to the medium used for the exchange.

## Supporting Information

Methods S1 **Materials for supporting figures and tables.**
(DOCX)

Figure S1 **Schematic diagram of the tetracycline (Tet)-regulated adenoviral expression system.** We used two types of adenoviral vectors to express γPKC-GFP using the tetracycline (Tet)-regulated gene expression system. The first type of vector was constructed to express the tetracycline transactivator (tTA). Cell lines (HeLa and MEF cells) were infected with Ad-CMV-tTA, which expresses tTA under the control of the CMV promoter. Primary-cultured cerebellar Purkinje cells were infected with Ad-L7-tTA expressing tTA under the control of the L7 promoter, which induces gene expression in a PC-specific manner. The second type of vector, Ad-TetOp-target protein (GFP- and HT-fused proteins), encodes cDNA of the target protein under the control of the TetOp minimal promoter, which is transactivated by tTA. Upon co-infection with these two adenoviral vectors, tTA binds to the TetOp promoter and activates the transcription of the target protein. The expression of GFP- and HT-fused proteins is controlled by the promoter in the first adenoviral vector. In the presence of Tet, expression of the target protein is turned off, since Tet-bound tTA is unable to bind the TetOp promoter.
(TIF)

Figure S2 **Immunoblotting and LC3 immunostaining of HeLa cells transfected with LAMP2A- and Atg5-siRNA.** (*A*) Representative immunoblots of HeLa cells transfected with nontargeting (Non)-, LAMP2A- and Atg5-siRNAs, detected with anti-LAMP2A, LAMP2, Atg5, LC3 and β-tubulin antibodies. Cells were harvested and analyzed 3 days after siRNA transfection. The anti-Atg5 antibody detected an Atg5-Atg12 complex at about 55 kDa. The LC3 antibody detected LC3-I (19 kDa) and LC3-II (16 kDa). (*B*) Quantitative analyses of immunoblotting data shown in *A*. The amount of each protein was normalized to the amount of β-tubulin. * p<0.05, ** p<0.01 and *** p<0.001 vs cells treated with nontargeting-siRNA (unpaired t-test, n = 3 in LAMP2, n = 5 in other proteins). (*C*) Representative LC3 immunostaining of HeLa cells transfected with nontargeting- (left), LAMP2A- (center) and Atg5- (right) siRNAs. Bar = 20 µm.
(TIF)

Figure S3 **Lysosomal translocation of GAPDH-HT in cells with inhibited macroautophagy.** (*A*) Representative fluorescent images of GAPDH-HT 21 h after labeling with TMR-HT ligand in HeLa cells transfected with nontargeting-siRNA (left) and Atg5-siRNA (right). Bar = 20 µm. (*B*) Quantitative analyses of GAPDH-HT lysosomal translocation in HeLa cells transfected with nontargeting (Non)- and Atg5-siRNAs. Percentages of GAPDH-HT-dot-positive cells were not significantly affected by siRNA-mediated knockdown of Atg5 (unpaired t-test, n = 16). (*C*) Representative fluorescent images of GAPDH-HT in HeLa cells treated with vehicle (0.1% DMSO, 0.1% methanol, left) or 3-methyladenine (3-MA; 10 mM, right) taken 21 h after labeling with TMR-HT ligand. Bar = 20 µm. (*D*) Quantitative analyses of GAPDH-HT lysosomal translocation in HeLa cells treated with 3-MA. Percentages of GAPDH-HT dot-positive cells were not significantly affected by 3-MA (unpaired t-test, n = 12 for cells treated with vehicle, n = 8 for cells treated with 3-MA). (*E*) Representative GAPDH-HT fluorescence (left), LAMP2A immunostaining (center) and merged (right) images of Atg5-KO MEF cells 21 h after labeling with TMR-HT ligand. Dots of GAPDH-HT strongly colocalized with LAMP2A-positive lysosomes. Bar = 10 µm.
(TIF)

Figure S4 **Immunostaining of LC3 and LAMP2A in HeLa cells displaying GAPDH-HT dots in the presence or absence of CMA activators.** (*A*) Representative GAPDH-HT fluorescence (upper panels), LC3 immunostaining (center panels) and merged (lower panels) images of HeLa cells treated with vehicle (0.1% DMSO, 0.1% methanol), serum free medium (0.1% DMSO, 0.1% methanol), H_2_O_2_ (100 µM) or MPA (10 µM) 21 h after labeling with TMR-HT ligand. While LC3-positive dots that represent autophagosomes were distributed diffusely in the cytoplasm, GAPDH-HT dots accumulated in the perinuclear region in the absence or presence of CMA activators. Bar = 5 µm. (*B*) Higher magnification images of squares in merged images of serum (−) (upper) and H_2_O_2_ (lower) treatments. Although serum deprivation and H_2_O_2_ increased the number of LC3-positive dots, these dots rarely colocalized with GAPDH-HT dots, suggesting that GAPDH-HT dots do not result from macroautophagy. (*C*) Representative GAPDH-HT fluorescence (upper panels), LC3 immunostaining (center panels) and merged (lower panels) images of HeLa cells treated with vehicle, serum free medium, H_2_O_2_ or MPA 21 h after labeling with TMR-HT ligand. Bar = 5 µm. (*D*) Higher magnification images of squares in merged images of serum (−) (upper) and H_2_O_2_ (lower) treatments. GAPDH-HT dots colocalized with or were surrounded by LAMP2A-positive dots in the absence or presence of CMA activators, indicating that lysosomal translocation of GAPDH-HT is mediated by CMA.
(TIF)

Figure S5 **Lysosomal translocation of GAPDH-HT was inhibited by siRNA-mediated knockdown of LAMP2A in primary rat cortical neurons.** (*A*) Representative fluorescence images of GAPDH-HT 21 h after labeling with TMR-HT ligand in cortical neurons transfected with nontargeting-siRNA (left) or LAMP2A-siRNA (right). Bar = 5 µm. (*B*) Quantitative analyses of lysosomal translocation of GAPDH-HT in cortical neuron somata transfected with nontargeting (Non)- and LAMP2A-siRNA. Dots of GAPDH-HT in each soma were counted in the center image from the Z-stack. Numbers of GAPDH-HT dots were significantly decreased by siRNA-mediated LAMP2A-knockdown. ** p<0.001 vs cells treated with nontargeting-siRNA (unpaired *t*-test, n = 30). (*C*) Representative immunoblots of primary rat cortical neurons transfected with nontargeting (Non)- and LAMP2A-siRNA, detected with anti-LAMP2A and β-tubulin antibodies. Cells were harvested and analyzed 3 days after siRNA transfection. We confirmed that the amount of LAMP2A was strongly decreased by siRNA-mediated LAMP2A-knockdown in primary rat cortical neurons.
(TIF)

Figure S6 **Preferred interaction of mutant γPKC with Hsc70 by HT pull-down assay.** (*A*) Representative silver stained gel of proteins obtained by the HT pull-down assay. The HT pull-down assay was conducted using cell lysate from cerebellar primary cultures that expressed WT or mutant (S119P and G128D) γPKC-HT in PCs. Input and supernatant (Sup) indicate cell lysates before and after pull-down with HT ligand-conjugated resin, respectively. The arrow indicates the protein band that was more strongly detected in pull-down samples from cell lysates expressing mutant γPKC-HT than in the WT. (*B*) Representative immunoblots of pull-down, input (5%) and Sup (5%) samples obtained by HT pull-down assay from SH-SY5Y cells expressing WT and mutant γPKC-HT. Samples were subjected to SDS-PAGE with 8% acrylamide gel, followed by immunoblotting with anti-HT (for γPKC-HT,), anti-Hsc70 and anti-β-tubulin antibodies. Hsc70 was strongly detected in pulled down samples with mutant γPKC-HT, compared with WT γPKC-HT, suggesting preferred binding of Hsc70 with mutant γPKC. In contrast, β-tubulin was similarly detected in pull down samples with WT and mutant γPKC-HT. Since pulled down proteins that bound with HaloLink resin were not released from resin in principal (Fig. 4A), the amount of pulled down proteins was estimated the difference in the band densities between input and Sup. However, γPKC-HT was also detected in pull down samples. This would be reflected by the self-association of pulled down γPKC-HT with that left in lysates. (*C*) Representative immunoblots of pull-down, input (5%) and Sup (5%) samples obtained by HT pull-down assay from SH-SY5Y cells expressing Hsc70-HT and WT/mutant γPKC. Expression of corresponding proteins were detected with anti-HT (for Hsc70-HT), anti-γPKC and anti-β-tubulin antibodies. Stronger γPKC-immunoreacitve bands were detected in pulled down samples from mutant γPKC-coexpressing cells than WT γPKC-coexpressing cells. In pull-down samples from mutant γPKC-coexpressing cells, an additional lower band was detected, compared with samples from γPKC-coexpressing cells. This would represent unphosphorylated mutant γPKC, since mutant γPKC was less phosphorylated [17]. This suggests that Hsc70 binds preferably with unphosphorylated mutant γPKC.
(TIF)

Figure S7 **SCA14 mutant γPKC suppresses CMA activity in cultured cell lines.** (*A*) Representative fluorescent images of γPKC-GFP (upper panels) and GAPDH-HT (lower panels) in HeLa cells 21 h after labeling with TMR-HT ligand. HeLa cells coexpressed GAPDH-HT and WT (left), S119P (center) or G128D (right) mutant γPKC-GFP. Lysosomal translocation of GAPDH-HT was inhibited by mutant γPKC-GFP in cells with and without aggregation of mutant γPKC-GFP. Bar = 20 µm. (*B*) Quantitative analyses of GAPDH-HT lysosomal translocation in HeLa cells. Mutant γPKC-GFPs (S119P and G128D) significantly decreased the number of GAPDH-HT dots (* p<0.01, ** p<0.001 vs cells expressing WT γPKC-GFP, n = 4, unpaired t-test), while there was no difference in lysosomal translocation between cells expressing GFP and WT γPKC-GFP. (*C*) Representative immunoblots of SH-SY5Y cells expressing WT and two mutant (S119P and G128D) forms of γPKC-GFP, detected with anti-GFP, anti-GAPDH, anti-MEF2D and anti-α-tubulin antibodies. Cells were harvested and analyzed 24 h after transduction with γPKC-GFP expression adenovirus. (*D*) Quantitative analyses of immunoblotting data shown in *C*. The amounts of GAPDH and MEF2D were normalized to the amount of α-tubulin. ** p<0.01 and *** p<0.001 vs cells expressing WT γPKC-GFP (unpaired t-test, n = 5). (*E*) Representative immunoblots of HeLa cells transfected with nontargeting (Non)- and LAMP2A-siRNAs, detected with anti-LAMP2A, GAPDH and β-tubulin antibodies. Cells were harvested and analyzed 3 days after siRNA transfection. (*F*) Quantitative analyses of immunoblotting data shown in *E* (n = 5). The amount of GAPDH was normalized to the amount of β-tubulin.
(TIF)

Table S1 **Results of 2-color FRAP analysis in primary cultured PCs expressing γPKC-GFP and HT-fused proteins (Hsc70-HT, HT, GAPDH-HT).** FRAP analysis was conducted immediately after labeling with TMR-HT ligand in PC somata not displaying aggregation of mutant γPKC-GFP. The half time of fluorescence recovery is inversely correlated with the mobility of a GFP- or HT-fused protein. The recovery half time of mutant γPKC-GFP was markedly longer than that of WT γPKC-GFP, probably due to oligomer formation [18]. The recovery half time of Hsc70-HT was significantly prolonged by coexpression with mutant γPKC-GFP, while the recovery half time of HT alone was not affected by the presence of mutant γPKC-GFP. These findings suggest that the mobility of Hsc70 is decreased due to a strong interaction with mutant γPKC in living PCs. On the contrary, the recovery half time of GAPDH-HT was significantly shortened by the presence of G128D mutant γPKC-GFP. * p<0.05, ** p<0.01, *** p<0.001 vs WT γPKC-GFP-expressing cells, unpaired t-test.
(TIF)

Table S2 **Results of RICS analysis in primary cultured PCs expressing γPKC-GFP and HT-fused proteins (Hsc70-HT, HT).** RICS analysis was conducted immediately after labeling with TMR-HT ligand in PC somata not displaying aggregation of mutant γPKC-GFP. The diffusion coefficient represents the mobility of GFP- and HT-fused proteins. The diffusion coefficient of mutant γPKC-GFP was markedly decreased from that of WT γPKC-GFP, probably due to oligomer formation [18]. The diffusion coefficient of Hsc70-HT was slightly but significantly decreased by coexpression of mutant γPKC-GFP, while the diffusion coefficient of HT was not affected by mutant γPKC-GFP, suggesting that the mobility of Hsc70 was reduced in the presence of mutant γPKC. Relative cross-correlation represents the percentage of fluorescent molecules that bind to molecules labeled with the other fluorophore. The relative cross-correlations of mutant γPKC-GFP and Hsc70-HT both significantly increased, compared with cells coexpressing WT γPKC-GFP and Hsc70-HT. These findings suggest that mutant γPKC strongly interacts with Hsc70 and reduces its mobility in living PCs. * p<0.05, ** p<0.01, *** p<0.001 vs WT γPKC-GFP-expressing cells, unpaired t-test.
(TIF)
