# Correction: Establishment of a Novel Fluorescence-Based Method to Evaluate Chaperone-Mediated Autophagy in a Single Neuron

**DOI:** 10.1371/journal.pone.0109006

**Published:** 2014-09-17

**Authors:** The PLOS ONE Staff

Figure S4 is incorrect. The second image in the first column of Figure S4C is from the incorrect cell. Please view the correct Figure S4 below.

## Supporting Information

Figure S4 **Immunostaining of LC3 and LAMP2A in HeLa cells displaying GAPDH-HT dots in the presence or absence of CMA activators.** (*A*) Representative GAPDH-HT fluorescence (upper panels), LC3 immunostaining (center panels) and merged (lower panels) images of HeLa cells treated with vehicle (0.1% DMSO, 0.1% methanol), serum free medium (0.1% DMSO, 0.1% methanol), H_2_O_2_ (100 µM) or MPA (10 µM) 21 h after labeling with TMR-HT ligand. While LC3-positive dots that represent autophagosomes were distributed diffusely in the cytoplasm, GAPDH-HT dots accumulated in the perinuclear region in the absence or presence of CMA activators. Bar  =  5 µm. (*B*) Higher magnification images of squares in merged images of serum (−) (upper) and H_2_O_2_ (lower) treatments. Although serum deprivation and H_2_O_2_ increased the number of LC3-positive dots, these dots rarely colocalized with GAPDH-HT dots, suggesting that GAPDH-HT dots do not result from macroautophagy. (*C*) Representative GAPDH-HT fluorescence (upper panels), LC3 immunostaining (center panels) and merged (lower panels) images of HeLa cells treated with vehicle, serum free medium, H_2_O_2_ or MPA 21 h after labeling with TMR-HT ligand. Bar  =  5 µm. (*D*) Higher magnification images of squares in merged images of serum (−) (upper) and H_2_O_2_ (lower) treatments. GAPDH-HT dots colocalized with or were surrounded by LAMP2A-positive dots in the absence or presence of CMA activators, indicating that lysosomal translocation of GAPDH-HT is mediated by CMA.(TIF)
(TIF)
