# Supplementary material for: Establishment of a Novel Fluorescence-Based Method to Evaluate Chaperone-Mediated Autophagy in a Single Neuron
Source: PLoS One. 2012 Feb 7;7(2):e31232. doi: 10.1371/journal.pone.0031232 (PMC3280339; doi:10.1371/journal.pone.0031232)
Supplement: Methods S1 — Materials for supporting figures and tables. (DOCX) [file pone.0031232.s001.docx]

*Methods S1. Materials for supporting figures and tables*

F-12 medium, anti-LC3 rabbit polyclonal, anti-Atg5 rabbit polyclonal and anti-α- and β-tubulin mouse monoclonal antibodies and 3-methyladenine were obtained from Sigma-Aldrich. Anti-GFP mouse monoclonal antibody was obtained from Nakalai Tesque (Kyoto, Japan). Horseradish peroxidase (HRP)-conjugated goat anti-mouse, anti-rabbit IgG and anti-mouse IgM antibodies were from Jackson ImmunoResearch Laboratories (West Grove, PA). Anti-LC3 rabbit polyclonal antibody for immunostaining was from MBL (Nagoya, Japan). Anti-γPKC rabbit polyclonal antibody was from Santa Cruz Biotechnology (Santa Cruz, CA). Anti-HaloTag rabbit polyclonal antibody was from Promega. Anti-Hsc70 mouse monoclonal IgM antibody was from AbCam. Anti-GAPDH mouse monoclonal antibody was from Millipore (Billerica, MA). Anti-MEF2D antibody was from BD Biosciences.
