# Supplementary figures and images for: Establishment of a Novel Fluorescence-Based Method to Evaluate Chaperone-Mediated Autophagy in a Single Neuron
Source: PLoS One. 2012 Feb 7;7(2):e31232. doi: 10.1371/journal.pone.0031232 (PMC3280339; doi:10.1371/journal.pone.0031232)

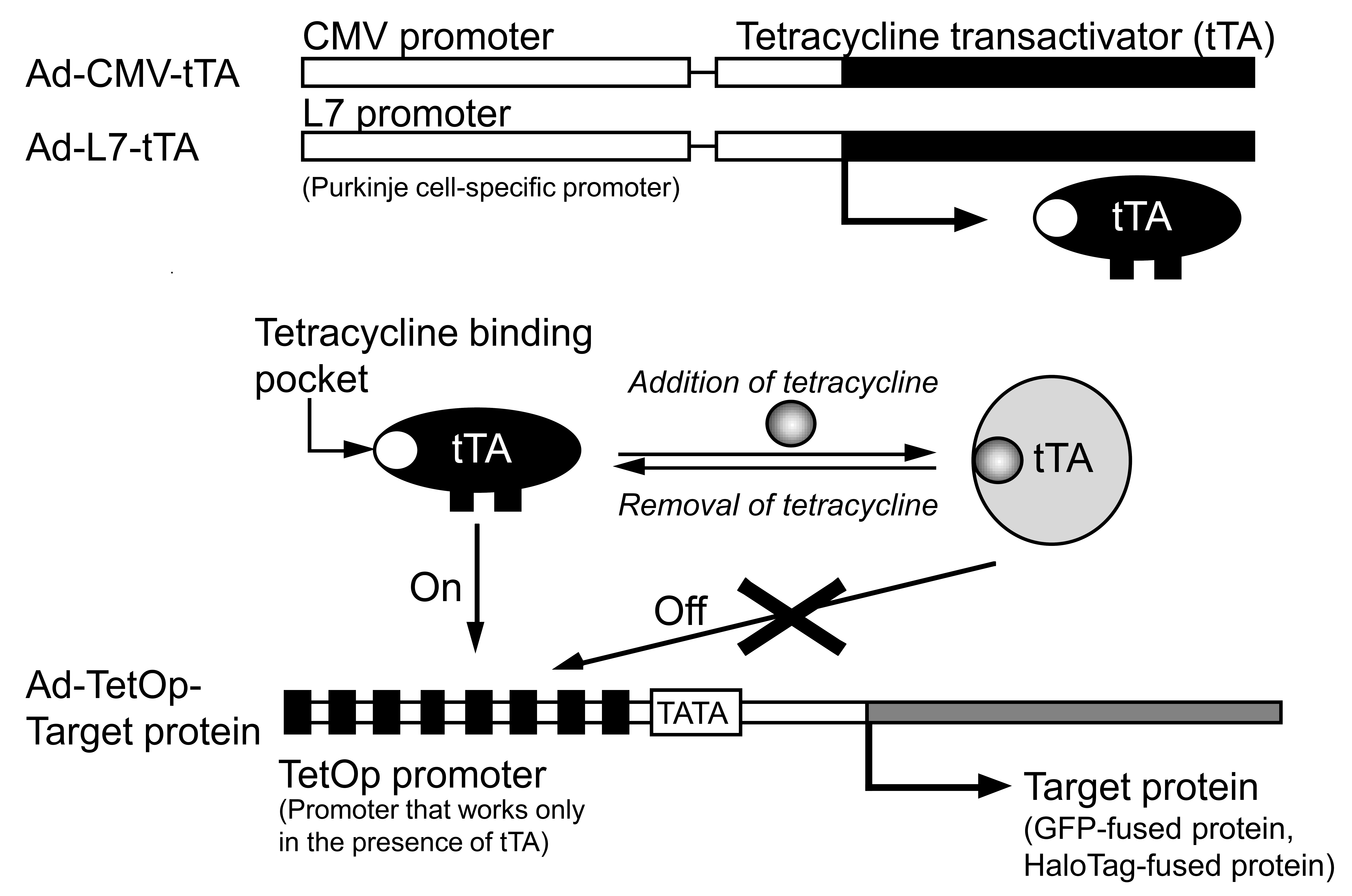

Supplement: Figure S1 — Schematic diagram of the tetracycline (Tet)-regulated adenoviral expression system. We used two types of adenoviral vectors to express γPKC-GFP using the tetracycline (Tet)-regulated gene expression system. The first type of vector was constructed to express the tetracycline transactivator (tTA). Cell lines (HeLa and MEF cells) were infected with Ad-CMV-tTA, which expresses tTA under the control of the CMV promoter. Primary-cultured cerebellar Purkinje cells were infected with Ad-L7-tTA expressing tTA under the control of the L7 promoter, which induces gene expression in a PC-specific manner. The second type of vector, Ad-TetOp-target protein (GFP- and HT-fused proteins), encodes cDNA of the target protein under the control of the TetOp minimal promoter, which is transactivated by tTA. Upon co-infection with these two adenoviral vectors, tTA binds to the TetOp promoter and activates the transcription of the target protein. The expression of GFP- and HT-fused proteins is controlled by the promoter in the first adenoviral vector. In the presence of Tet, expression of the target protein is turned off, since Tet-bound tTA is unable to bind the TetOp promoter. (TIF) [file pone.0031232.s002.tif]

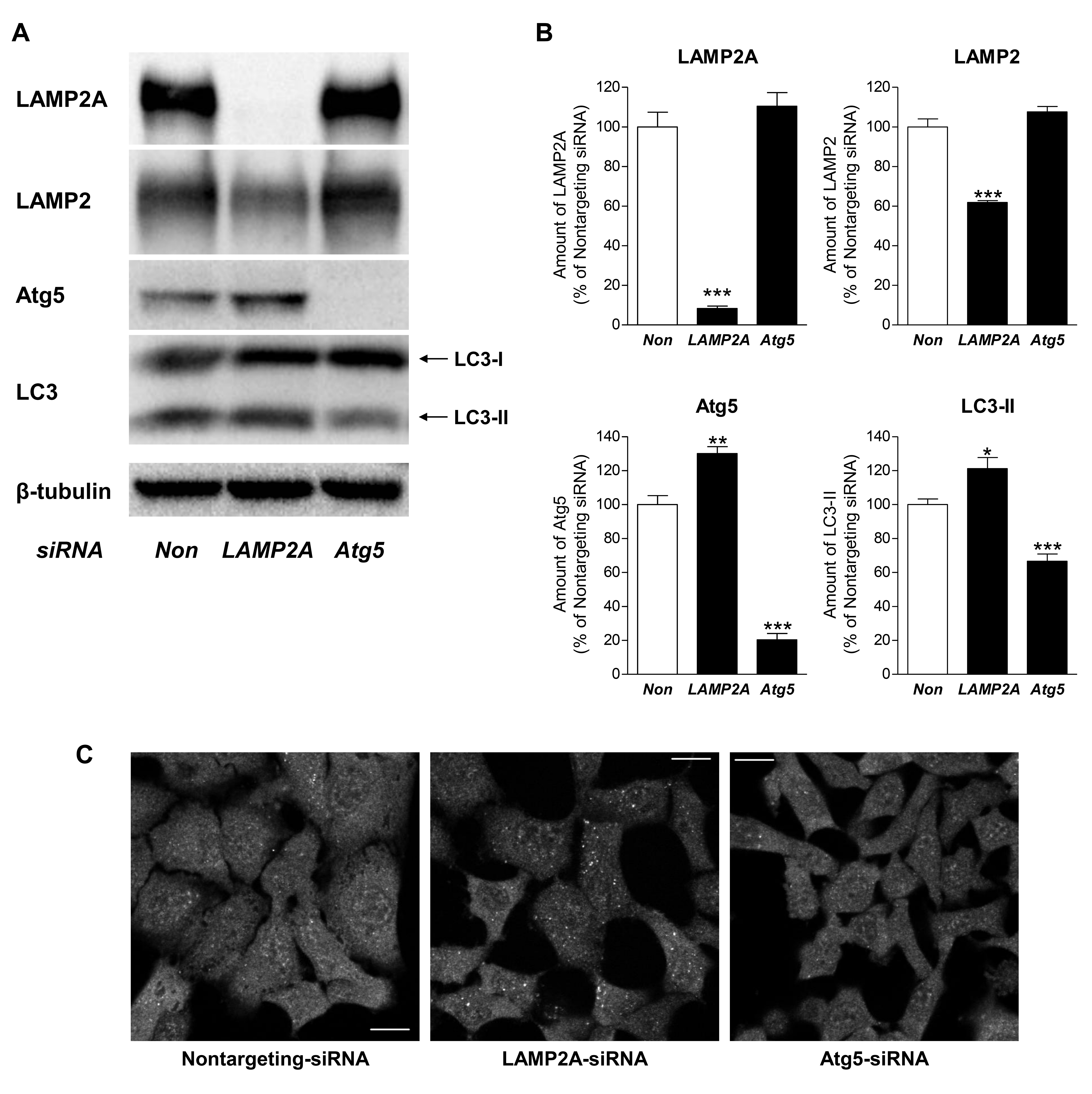

Supplement: Figure S2 — Immunoblotting and LC3 immunostaining of HeLa cells transfected with LAMP2A- and Atg5-siRNA. (A) Representative immunoblots of HeLa cells transfected with nontargeting (Non)-, LAMP2A- and Atg5-siRNAs, detected with anti-LAMP2A, LAMP2, Atg5, LC3 and β-tubulin antibodies. Cells were harvested and analyzed 3 days after siRNA transfection. The anti-Atg5 antibody detected an Atg5-Atg12 complex at about 55 kDa. The LC3 antibody detected LC3-I (19 kDa) and LC3-II (16 kDa). (B) Quantitative analyses of immunoblotting data shown in A. The amount of each protein was normalized to the amount of β-tubulin. * p<0.05, ** p<0.01 and *** p<0.001 vs cells treated with nontargeting-siRNA (unpaired t-test, n = 3 in LAMP2, n = 5 in other proteins). (C) Representative LC3 immunostaining of HeLa cells transfected with nontargeting- (left), LAMP2A- (center) and Atg5- (right) siRNAs. Bar = 20 µm. (TIF) [file pone.0031232.s003.tif]

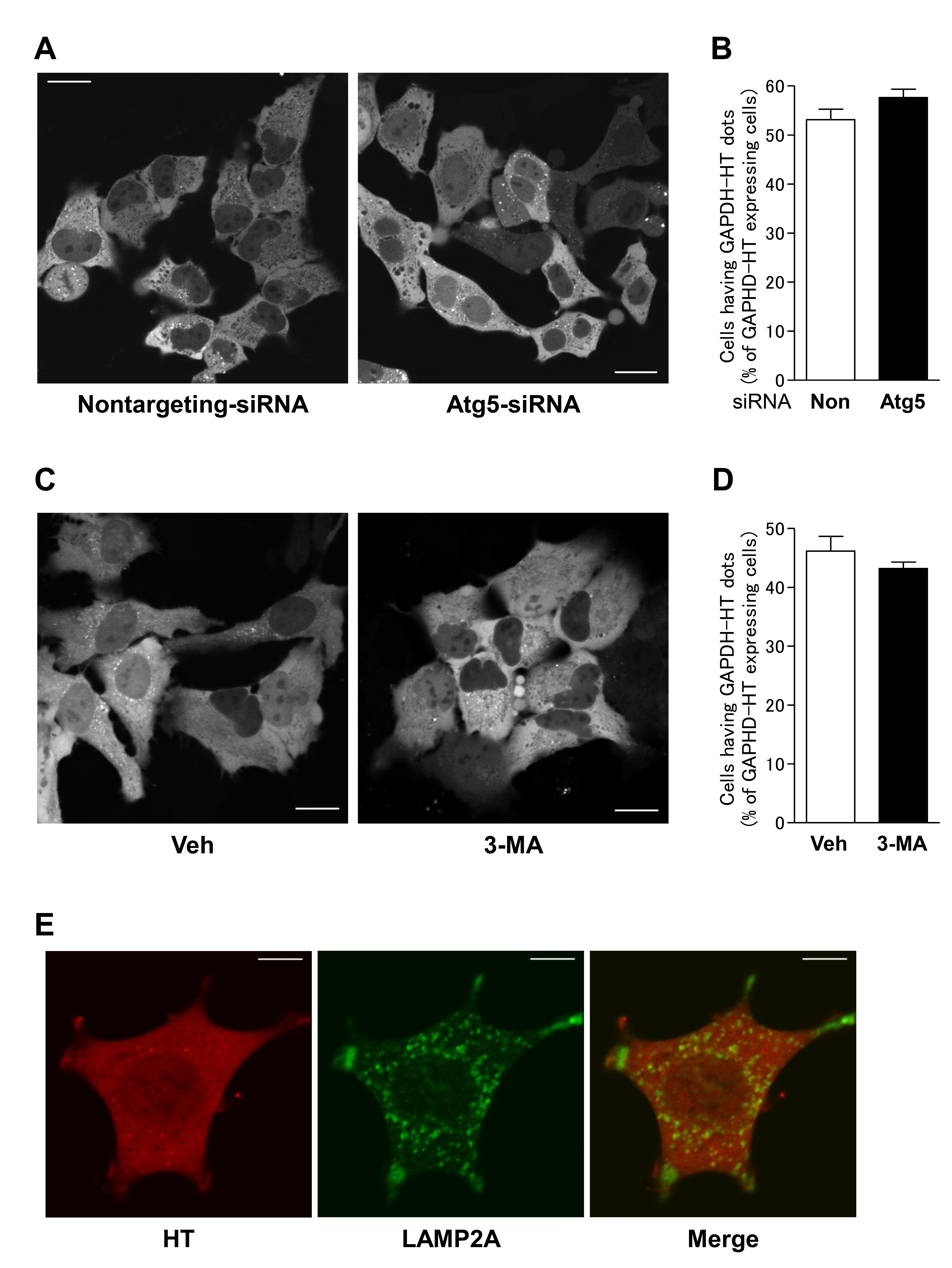

Supplement: Figure S3 — Lysosomal translocation of GAPDH-HT in cells with inhibited macroautophagy. (A) Representative fluorescent images of GAPDH-HT 21 h after labeling with TMR-HT ligand in HeLa cells transfected with nontargeting-siRNA (left) and Atg5-siRNA (right). Bar = 20 µm. (B) Quantitative analyses of GAPDH-HT lysosomal translocation in HeLa cells transfected with nontargeting (Non)- and Atg5-siRNAs. Percentages of GAPDH-HT-dot-positive cells were not significantly affected by siRNA-mediated knockdown of Atg5 (unpaired t-test, n = 16). (C) Representative fluorescent images of GAPDH-HT in HeLa cells treated with vehicle (0.1% DMSO, 0.1% methanol, left) or 3-methyladenine (3-MA; 10 mM, right) taken 21 h after labeling with TMR-HT ligand. Bar = 20 µm. (D) Quantitative analyses of GAPDH-HT lysosomal translocation in HeLa cells treated with 3-MA. Percentages of GAPDH-HT dot-positive cells were not significantly affected by 3-MA (unpaired t-test, n = 12 for cells treated with vehicle, n = 8 for cells treated with 3-MA). (E) Representative GAPDH-HT fluorescence (left), LAMP2A immunostaining (center) and merged (right) images of Atg5-KO MEF cells 21 h after labeling with TMR-HT ligand. Dots of GAPDH-HT strongly colocalized with LAMP2A-positive lysosomes. Bar = 10 µm. (TIF) [file pone.0031232.s004.tif]

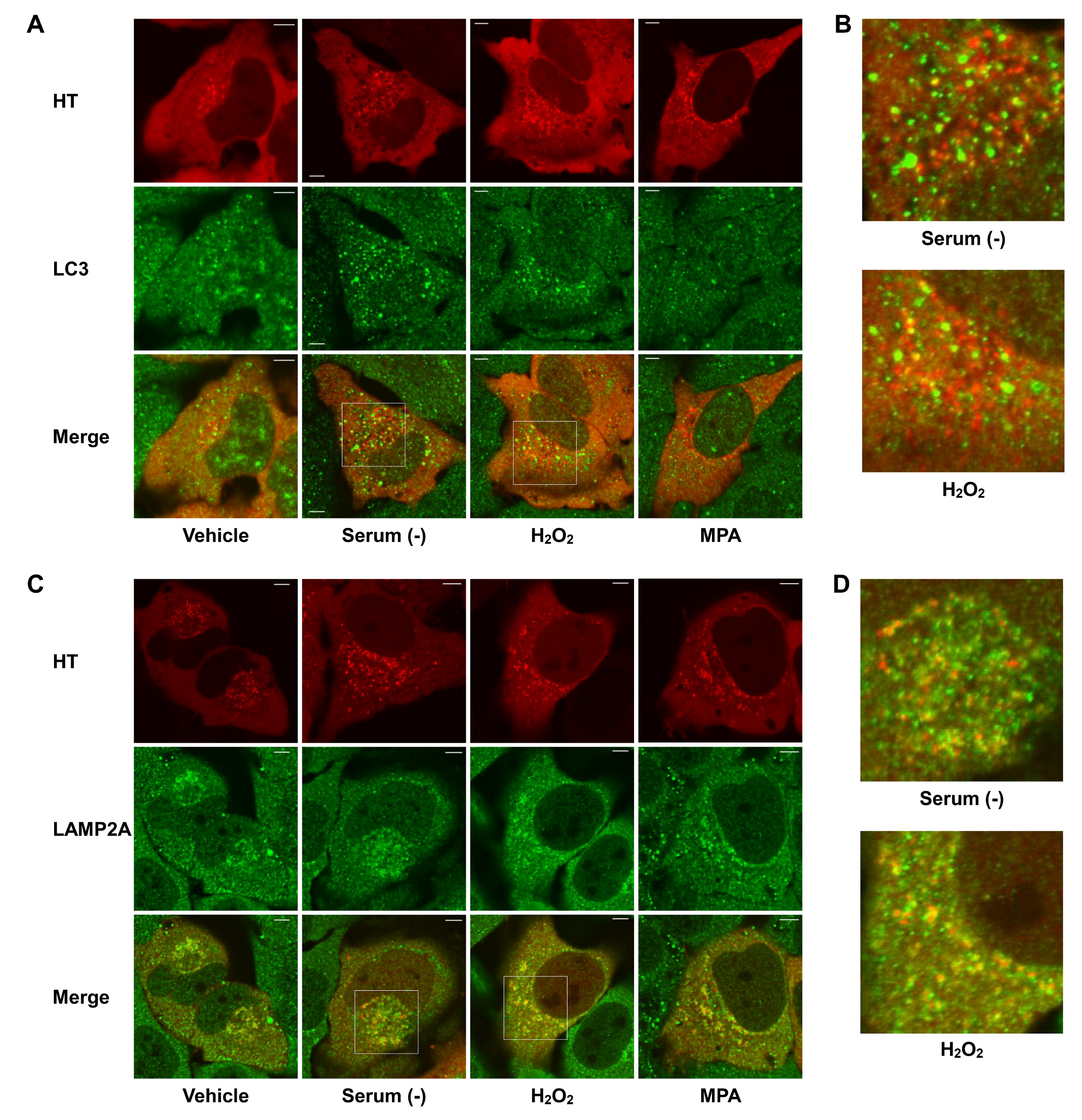

Supplement: Figure S4 — Immunostaining of LC3 and LAMP2A in HeLa cells displaying GAPDH-HT dots in the presence or absence of CMA activators. (A) Representative GAPDH-HT fluorescence (upper panels), LC3 immunostaining (center panels) and merged (lower panels) images of HeLa cells treated with vehicle (0.1% DMSO, 0.1% methanol), serum free medium (0.1% DMSO, 0.1% methanol), H2O2 (100 µM) or MPA (10 µM) 21 h after labeling with TMR-HT ligand. While LC3-positive dots that represent autophagosomes were distributed diffusely in the cytoplasm, GAPDH-HT dots accumulated in the perinuclear region in the absence or presence of CMA activators. Bar = 5 µm. (B) Higher magnification images of squares in merged images of serum (−) (upper) and H2O2 (lower) treatments. Although serum deprivation and H2O2 increased the number of LC3-positive dots, these dots rarely colocalized with GAPDH-HT dots, suggesting that GAPDH-HT dots do not result from macroautophagy. (C) Representative GAPDH-HT fluorescence (upper panels), LC3 immunostaining (center panels) and merged (lower panels) images of HeLa cells treated with vehicle, serum free medium, H2O2 or MPA 21 h after labeling with TMR-HT ligand. Bar = 5 µm. (D) Higher magnification images of squares in merged images of serum (−) (upper) and H2O2 (lower) treatments. GAPDH-HT dots colocalized with or were surrounded by LAMP2A-positive dots in the absence or presence of CMA activators, indicating that lysosomal translocation of GAPDH-HT is mediated by CMA. (TIF) [file pone.0031232.s005.tif]

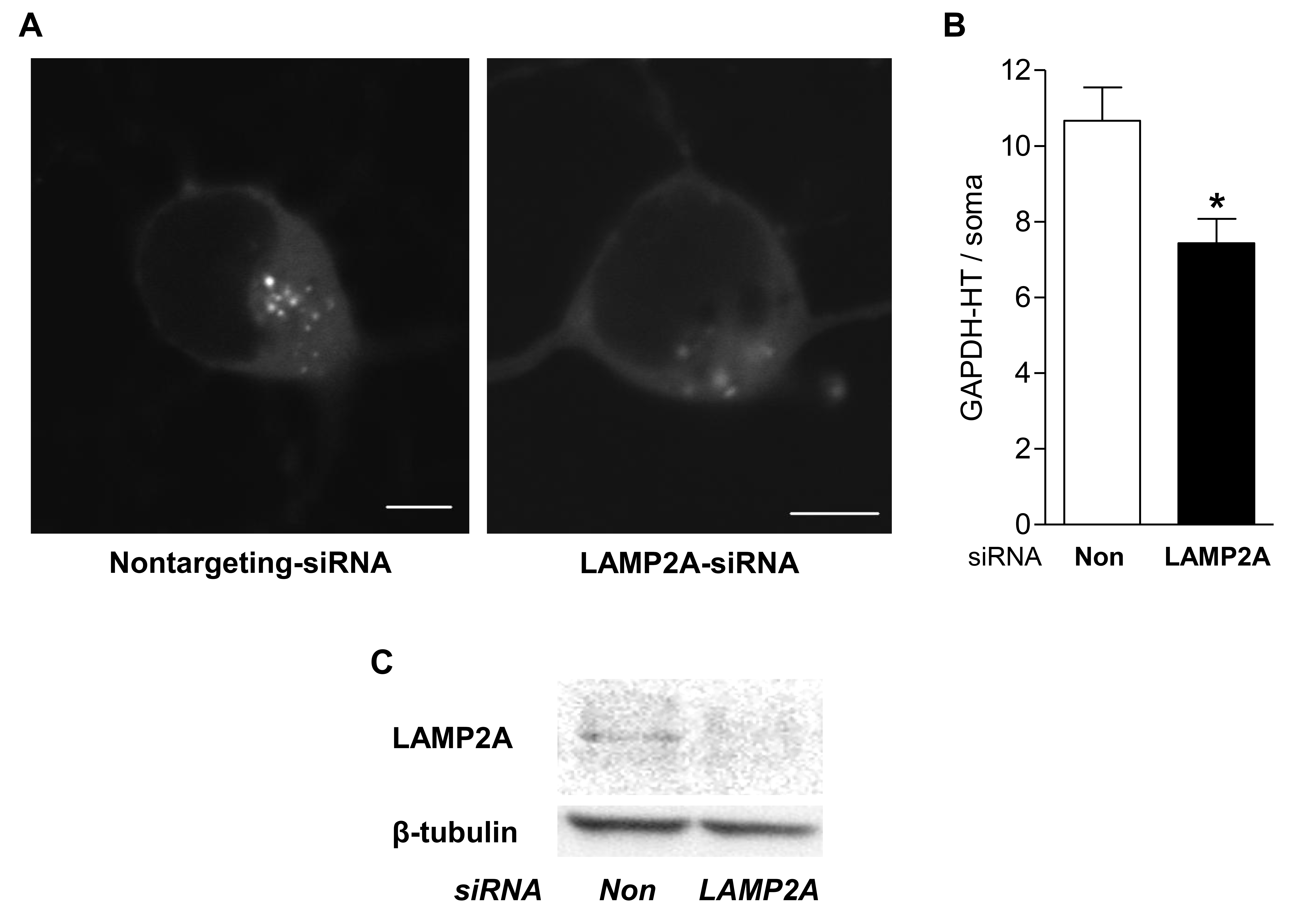

Supplement: Figure S5 — Lysosomal translocation of GAPDH-HT was inhibited by siRNA-mediated knockdown of LAMP2A in primary rat cortical neurons. (A) Representative fluorescence images of GAPDH-HT 21 h after labeling with TMR-HT ligand in cortical neurons transfected with nontargeting-siRNA (left) or LAMP2A-siRNA (right). Bar = 5 µm. (B) Quantitative analyses of lysosomal translocation of GAPDH-HT in cortical neuron somata transfected with nontargeting (Non)- and LAMP2A-siRNA. Dots of GAPDH-HT in each soma were counted in the center image from the Z-stack. Numbers of GAPDH-HT dots were significantly decreased by siRNA-mediated LAMP2A-knockdown. ** p<0.001 vs cells treated with nontargeting-siRNA (unpaired t-test, n = 30). (C) Representative immunoblots of primary rat cortical neurons transfected with nontargeting (Non)- and LAMP2A-siRNA, detected with anti-LAMP2A and β-tubulin antibodies. Cells were harvested and analyzed 3 days after siRNA transfection. We confirmed that the amount of LAMP2A was strongly decreased by siRNA-mediated LAMP2A-knockdown in primary rat cortical neurons. (TIF) [file pone.0031232.s006.tif]

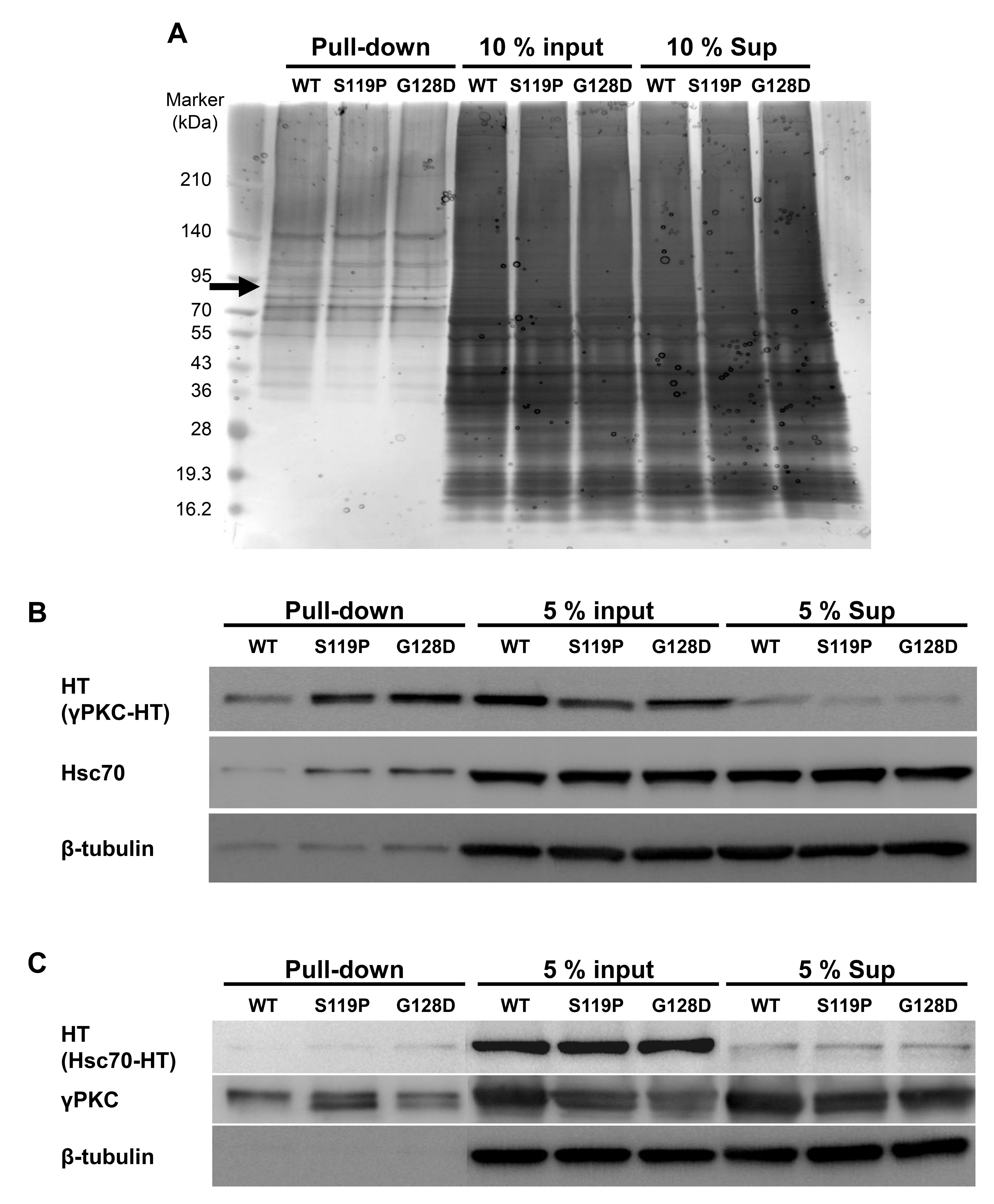

Supplement: Figure S6 — Preferred interaction of mutant γPKC with Hsc70 by HT pull-down assay. (A) Representative silver stained gel of proteins obtained by the HT pull-down assay. The HT pull-down assay was conducted using cell lysate from cerebellar primary cultures that expressed WT or mutant (S119P and G128D) γPKC-HT in PCs. Input and supernatant (Sup) indicate cell lysates before and after pull-down with HT ligand-conjugated resin, respectively. The arrow indicates the protein band that was more strongly detected in pull-down samples from cell lysates expressing mutant γPKC-HT than in the WT. (B) Representative immunoblots of pull-down, input (5%) and Sup (5%) samples obtained by HT pull-down assay from SH-SY5Y cells expressing WT and mutant γPKC-HT. Samples were subjected to SDS-PAGE with 8% acrylamide gel, followed by immunoblotting with anti-HT (for γPKC-HT,), anti-Hsc70 and anti-β-tubulin antibodies. Hsc70 was strongly detected in pulled down samples with mutant γPKC-HT, compared with WT γPKC-HT, suggesting preferred binding of Hsc70 with mutant γPKC. In contrast, β-tubulin was similarly detected in pull down samples with WT and mutant γPKC-HT. Since pulled down proteins that bound with HaloLink resin were not released from resin in principal (Fig. 4A), the amount of pulled down proteins was estimated the difference in the band densities between input and Sup. However, γPKC-HT was also detected in pull down samples. This would be reflected by the self-association of pulled down γPKC-HT with that left in lysates. (C) Representative immunoblots of pull-down, input (5%) and Sup (5%) samples obtained by HT pull-down assay from SH-SY5Y cells expressing Hsc70-HT and WT/mutant γPKC. Expression of corresponding proteins were detected with anti-HT (for Hsc70-HT), anti-γPKC and anti-β-tubulin antibodies. Stronger γPKC-immunoreacitve bands were detected in pulled down samples from mutant γPKC-coexpressing cells than WT γPKC-coexpressing cells. In pull-down samples from mutant γPKC-coexpressi [file pone.0031232.s007.tif]

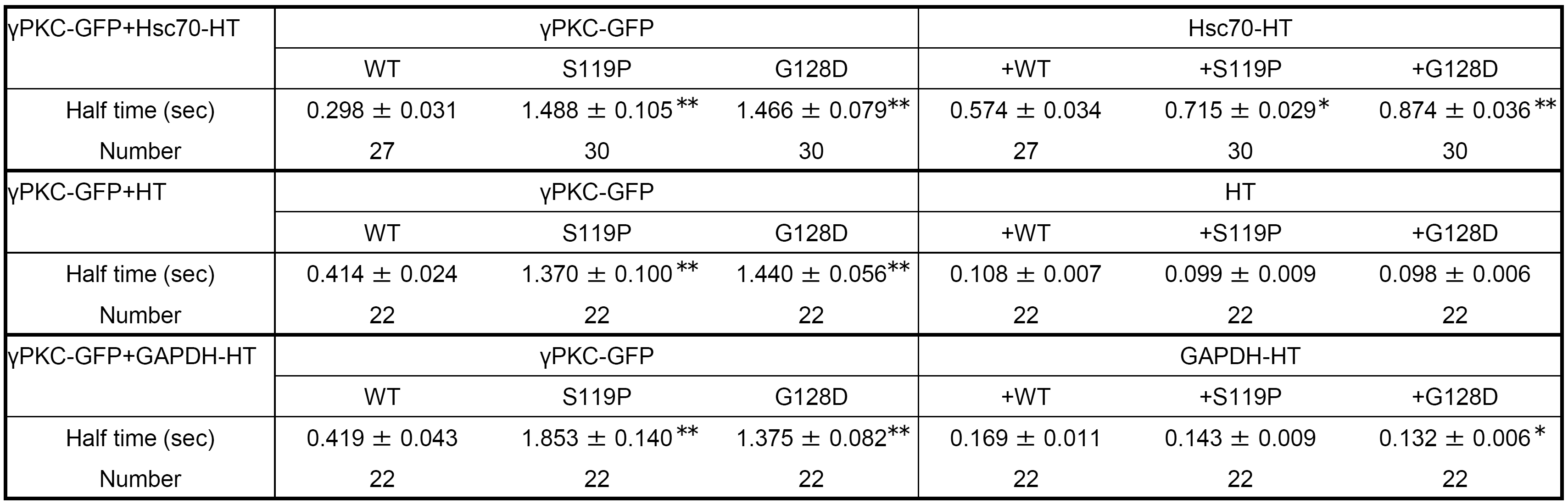

Supplement: Table S1 — Results of 2-color FRAP analysis in primary cultured PCs expressing γPKC-GFP and HT-fused proteins (Hsc70-HT, HT, GAPDH-HT). FRAP analysis was conducted immediately after labeling with TMR-HT ligand in PC somata not displaying aggregation of mutant γPKC-GFP. The half time of fluorescence recovery is inversely correlated with the mobility of a GFP- or HT-fused protein. The recovery half time of mutant γPKC-GFP was markedly longer than that of WT γPKC-GFP, probably due to oligomer formation [18]. The recovery half time of Hsc70-HT was significantly prolonged by coexpression with mutant γPKC-GFP, while the recovery half time of HT alone was not affected by the presence of mutant γPKC-GFP. These findings suggest that the mobility of Hsc70 is decreased due to a strong interaction with mutant γPKC in living PCs. On the contrary, the recovery half time of GAPDH-HT was significantly shortened by the presence of G128D mutant γPKC-GFP. * p<0.05, ** p<0.01, *** p<0.001 vs WT γPKC-GFP-expressing cells, unpaired t-test. (TIF) [file pone.0031232.s009.tif]

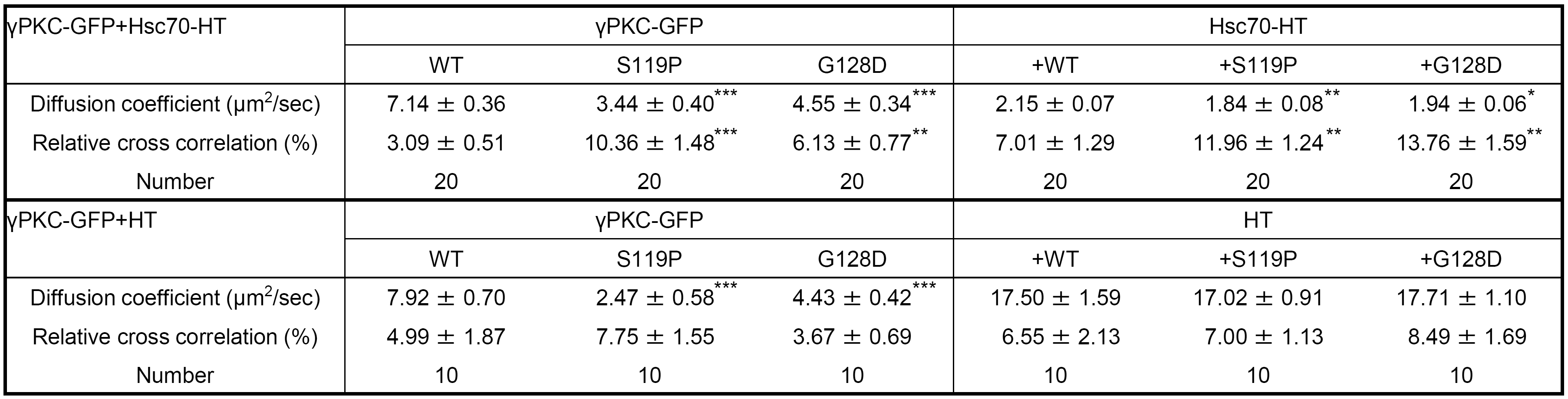

Supplement: Table S2 — Results of RICS analysis in primary cultured PCs expressing γPKC-GFP and HT-fused proteins (Hsc70-HT, HT). RICS analysis was conducted immediately after labeling with TMR-HT ligand in PC somata not displaying aggregation of mutant γPKC-GFP. The diffusion coefficient represents the mobility of GFP- and HT-fused proteins. The diffusion coefficient of mutant γPKC-GFP was markedly decreased from that of WT γPKC-GFP, probably due to oligomer formation [18]. The diffusion coefficient of Hsc70-HT was slightly but significantly decreased by coexpression of mutant γPKC-GFP, while the diffusion coefficient of HT was not affected by mutant γPKC-GFP, suggesting that the mobility of Hsc70 was reduced in the presence of mutant γPKC. Relative cross-correlation represents the percentage of fluorescent molecules that bind to molecules labeled with the other fluorophore. The relative cross-correlations of mutant γPKC-GFP and Hsc70-HT both significantly increased, compared with cells coexpressing WT γPKC-GFP and Hsc70-HT. These findings suggest that mutant γPKC strongly interacts with Hsc70 and reduces its mobility in living PCs. * p<0.05, ** p<0.01, *** p<0.001 vs WT γPKC-GFP-expressing cells, unpaired t-test. (TIF) [file pone.0031232.s010.tif]
