# Supplementary figures and images for: Correction: Establishment of a Novel Fluorescence-Based Method to Evaluate Chaperone-Mediated Autophagy in a Single Neuron
Source: PLoS One. 2014 Sep 17;9(9):e109006. doi: 10.1371/journal.pone.0109006 (PMC4168230; doi:10.1371/journal.pone.0109006)

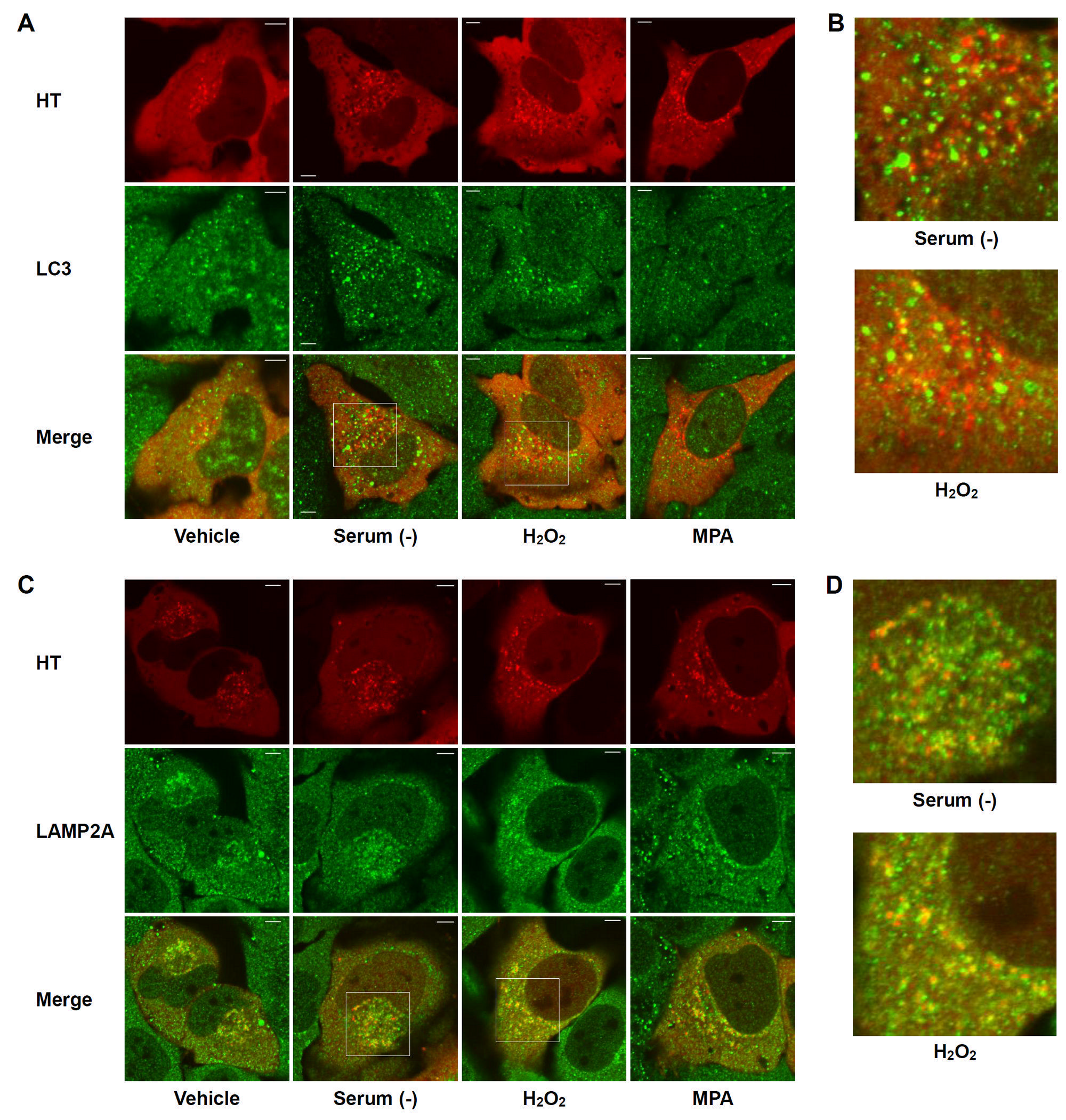

Supplement: Figure S4 — Immunostaining of LC3 and LAMP2A in HeLa cells displaying GAPDH-HT dots in the presence or absence of CMA activators. (A) Representative GAPDH-HT fluorescence (upper panels), LC3 immunostaining (center panels) and merged (lower panels) images of HeLa cells treated with vehicle (0.1% DMSO, 0.1% methanol), serum free medium (0.1% DMSO, 0.1% methanol), H2O2 (100 µM) or MPA (10 µM) 21 h after labeling with TMR-HT ligand. While LC3-positive dots that represent autophagosomes were distributed diffusely in the cytoplasm, GAPDH-HT dots accumulated in the perinuclear region in the absence or presence of CMA activators. Bar = 5 µm. (B) Higher magnification images of squares in merged images of serum (−) (upper) and H2O2 (lower) treatments. Although serum deprivation and H2O2 increased the number of LC3-positive dots, these dots rarely colocalized with GAPDH-HT dots, suggesting that GAPDH-HT dots do not result from macroautophagy. (C) Representative GAPDH-HT fluorescence (upper panels), LC3 immunostaining (center panels) and merged (lower panels) images of HeLa cells treated with vehicle, serum free medium, H2O2 or MPA 21 h after labeling with TMR-HT ligand. Bar = 5 µm. (D) Higher magnification images of squares in merged images of serum (−) (upper) and H2O2 (lower) treatments. GAPDH-HT dots colocalized with or were surrounded by LAMP2A-positive dots in the absence or presence of CMA activators, indicating that lysosomal translocation of GAPDH-HT is mediated by CMA.(TIF) (TIF) [file pone.0109006.s001.tif]
